# International Partnerships in AI-Driven Healthcare: Opportunities and Challenges for Advancing the UN Sustainable Development Goals—A Perspective

**DOI:** 10.3390/healthcare13162053

**Published:** 2025-08-20

**Authors:** Tao Yun, Le Zhang

**Affiliations:** 1 International Science and Technology Cooperation Center, Ministry of Science and Technology of the People’s Republic of China, Beijing 100862, China; 2 School of Information and Communication Engineering, University of Electronic Science and Technology of China, Chengdu 611731, China; zhangleuestc@gmail.com

**Keywords:** artificial intelligence, healthcare, international partnership, sustainable development

## Abstract

Artificial Intelligence (AI) is reshaping global healthcare systems by offering innovative solutions to improve diagnostic accuracy, optimize treatment planning, and enhance public health management. This article provides a structured perspective on the role of international partnerships in accelerating the adoption of AI-driven healthcare, with a focus on advancing the United Nations Sustainable Development Goals (SDGs), particularly SDG 3 (Good Health and Well-being). Drawing on representative global initiatives, the paper explores best practices in technology transfer, ethical data sharing, and capacity building—key enablers for inclusive and equitable AI healthcare adoption. It further analyzes common challenges such as digital infrastructure gaps, regulatory fragmentation, and global inequities in data and talent. Through a synthesis of recent collaborations and policy frameworks, this article offers actionable insights for fostering global alliances that bridge innovation with healthcare accessibility.

## 1. Introduction

The United Nations’ Sustainable Development Goals (SDGs) have laid out an ambitious vision for a healthier and more equitable world [1]. Goal 3, which aims to ensure healthy lives and promote well-being for all at all ages [2], is crucial to achieving many of the other SDGs. While remarkable progress has been made in several areas, significant gaps remain, particularly in the equitable distribution of healthcare services across different parts of the world [3]. AI, with its transformative potential in healthcare, presents a promising avenue to address these disparities [4]. However, to maximize its impact, international cooperation and partnerships are essential [5].

Historically, healthcare challenges have been addressed through a combination of public health programs, advancements in medical technology, and policy-driven initiatives aimed at improving access and equity. Governments and international organizations, such as the World Health Organization (WHO), have played pivotal roles in tackling widespread health issues, particularly through vaccination campaigns, disease eradication efforts, and the establishment of universal health coverage frameworks [3]. Technological innovations, such as imaging systems, telemedicine platforms, and pharmaceutical breakthroughs, have revolutionized diagnostics and treatment, yet these solutions often remain limited to high-resource settings [4]. Non-governmental organizations (NGOs) and philanthropic initiatives have also contributed significantly, particularly in low-resource regions, by providing funding, expertise, and on-the-ground support for combating infectious diseases, maternal health issues, and malnutrition [6]. Despite these efforts, existing approaches have been constrained by fragmented healthcare systems, workforce shortages, and the inability to scale solutions effectively to underserved populations [7]. Furthermore, the reliance on traditional methods has often been insufficient in addressing the growing burden of chronic diseases, aging populations, and health emergencies, underscoring the need for more transformative and scalable solutions [8].

Artificial Intelligence (AI) refers to the simulation of human intelligence by machines, particularly computer systems, which are capable of performing tasks that typically require human cognition, such as learning, reasoning, problem-solving, and decision-making [9]. In its early stages, AI primarily focused on rule-based systems and expert systems designed to solve specific problems within defined parameters [10]. These traditional systems, while useful in constrained environments, often lacked adaptability and the ability to learn from new data. However, with the advent of machine learning and deep learning, AI has evolved into a dynamic and data-driven field. Modern AI systems can analyze vast and complex datasets, identify patterns, and make predictions with remarkable accuracy, surpassing human performance in many specialized tasks [11]. This transformation has positioned AI as a pivotal tool for addressing global challenges, including those outlined in the Sustainable Development Goals (SDGs).

**Figure 1:**
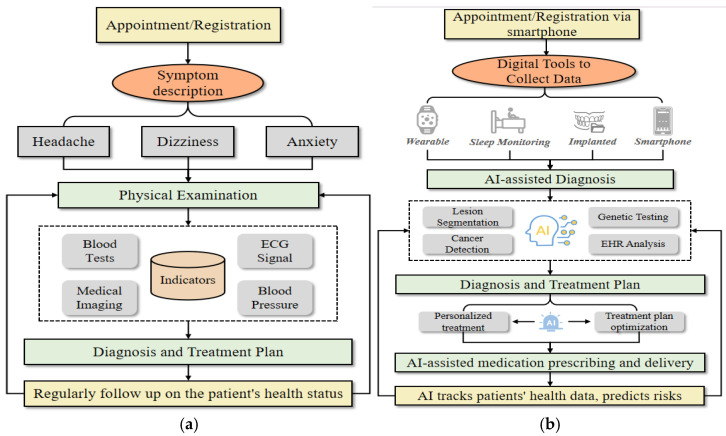
AI revolutionizes healthcare by replacing traditional, human-dependent processes with data-driven, efficient, and personalized care. (**a**) Traditional clinic procedure. (**b**) AI-based clinic procedure.

AI has made a significant impact on the healthcare field, revolutionizing traditional practices and creating new opportunities for more efficient and personalized care [12,13]. This shift has greatly influenced the way healthcare processes are carried out, from diagnosis to treatment and patient management. As illustrated in Figure 1, in traditional healthcare systems, the process is generally linear and heavily reliant on human expertise. The steps include appointment scheduling, symptom description, physical examinations, diagnosis and treatment planning, prescription of medications or treatments, and follow-up care. Doctors assess the patient’s condition based on their experience, laboratory tests, and imaging studies, which are then used to develop a treatment plan. While effective, this system can sometimes be limited by human error, inefficiencies, and a one-size-fits-all approach. In contrast, AI-driven healthcare systems leverage advanced technologies such as machine learning, computer vision, and natural language processing to enhance each stage of the process. From initial consultations to diagnosis, treatment planning, and patient monitoring, AI tools analyze large datasets to offer more precise, data-driven insights. AI can assist in diagnosing conditions from medical images, predicting disease progression, and even suggesting personalized treatment plans based on an individual’s unique health data. This integration of AI aims to improve efficiency, reduce human error, and enable more personalized, adaptive care. By automating routine tasks and providing intelligent insights, AI-based healthcare can offer quicker, more accurate, and tailored treatments compared to traditional methods.

**Figure 2:**
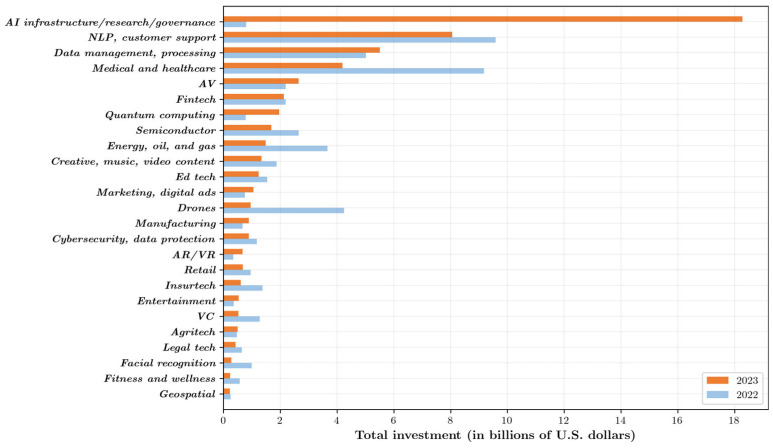
Private investment in Al by focus area, 2022 vs. 2023 (Source: Quid, 2023. Chart: 2024 Al index report).

AI offers significant advantages over traditional healthcare methods by improving accuracy, efficiency, and personalization. With its ability to analyze vast amounts of data, AI enhances diagnostic precision, reduces human error, and accelerates decision-making. It also enables personalized treatment plans tailored to individual patient needs, ultimately leading to better outcomes. These benefits, along with the potential for cost reduction and improved accessibility, have driven increased investment in AI-driven healthcare solutions, as stakeholders recognize the transformative potential of AI to address longstanding challenges in the industry. As illustrated in Figure 2, the healthcare sector has been a significant focus of AI investments, especially in countries like the United States, where a large proportion of AI funding is directed towards medical applications. By harnessing its ability to enhance diagnostic accuracy, streamline resource allocation, and personalize treatment, AI can address some of the most pressing issues in global healthcare [14]. For example, AI-powered imaging tools can identify diseases at earlier stages, while natural language processing systems can analyze patient records to provide actionable insights [4]. Moreover, AI can help mitigate the impact of workforce shortages by automating administrative and clinical tasks, freeing up healthcare professionals to focus on patient care [15]. Its capacity to integrate data from diverse sources—ranging from electronic health records to wearable devices—enables a holistic approach to managing chronic diseases and delivering tailored care [16]. In this rapidly changing landscape, AI is not merely a tool but a catalyst for innovation, bridging the gap between technological advancements and equitable access to healthcare services. Its pivotal role in addressing healthcare disparities positions AI as an indispensable enabler of the Sustainable Development Goals (SDGs), particularly SDG 3, which focuses on ensuring healthy lives and well-being for all [17].

AI for healthcare fosters international collaboration more effectively than traditional approaches due to its data-driven nature and scalable solutions. Unlike traditional healthcare, which often depends on local expertise and infrastructure, AI systems can leverage global datasets, algorithms, and tools that transcend geographic boundaries. By processing large and diverse datasets, AI creates more generalized, accurate, and adaptable solutions that can be shared across countries. This enables faster innovation, as countries with varying healthcare resources can collaborate, share knowledge, and pool data, improving AI model robustness. Moreover, international partnerships help overcome local challenges, such as shortages in medical professionals, and ensure that AI healthcare systems are developed with fairness and equity in mind, promoting better health outcomes worldwide.

This paper discusses the critical role of international partnerships and alliances in promoting AI-driven healthcare initiatives. These collaborations have the potential to address pressing challenges such as healthcare workforce shortages, disparities in access to care, and the growing demand for personalized healthcare services. By pooling resources, expertise, and technology, international partnerships can facilitate the development and deployment of AI solutions tailored to the unique needs of different regions. For example, partnerships can enable the co-creation of datasets that are more representative of diverse populations, thereby improving the fairness and generalizability of AI models. Additionally, joint initiatives can support the sharing of best practices and lessons learned, ensuring that low-resource settings benefit from innovations pioneered in high-resource environments. Such collaborations also provide opportunities for capacity building through training programs and knowledge transfer, empowering local healthcare professionals and organizations to leverage AI effectively. Furthermore, global alliances can play a pivotal role in addressing ethical and regulatory challenges, fostering trust and ensuring the responsible use of AI in healthcare. Through these efforts, international partnerships can bridge the gap between technological innovation and real-world impact, driving equitable access to quality healthcare services worldwide.

To structure this discussion, we focus on three core pillars of effective international collaboration in AI-driven healthcare: (1) technology transfer, which enables the adaptation and deployment of AI tools in local health systems; (2) ethical data sharing, which supports the development of representative and fair AI models; and (3) capacity building, which strengthens local institutions and human resources to implement and sustain AI solutions. These pillars form an interconnected framework through which international partnerships can help translate technological advancements into scalable, context-sensitive, and equitable healthcare outcomes. The following sections explore these elements in detail, supported by representative global initiatives and aligned with the broader objectives of the Sustainable Development Goals (SDGs).

## 2. AI in Healthcare: A Global Perspective

As mentioned above, AI technologies, including machine learning, natural language processing, and computer vision, have shown great promise in various healthcare domains, such as diagnostics, treatment planning, and health monitoring. For instance, AI algorithms have demonstrated capabilities comparable to human experts in detecting diseases from medical imaging, making early intervention possible in resource-limited settings [18]. Moreover, AI-driven predictive analytics can help manage public health crises, such as pandemics, by forecasting disease outbreaks and guiding decision-making processes [19].

Despite these advancements, the deployment of AI in healthcare remains highly uneven across countries, reflecting broader global disparities in technology adoption [20]. High-income nations often lead in adopting and integrating these technologies, driven by robust infrastructure, abundant data, and a well-trained workforce [21]. For example, a recent report highlights that over 70% of AI research publications and patents originate from high-income countries, leaving low- and middle-income nations (LMICs) with limited contributions [22]. Furthermore, high-income countries benefit from advanced digital infrastructure, with nearly 90% of their populations having access to the internet, compared to only 20% in least-developed countries [22]. This disparity provides high-income nations with a strong foundation to collect, analyze, and leverage data for AI healthcare solutions.

In contrast, LMICs face significant barriers that hinder the effective deployment of AI in healthcare. These include inadequate digital infrastructure, limited access to high-quality and representative datasets, and a shortage of AI expertise among healthcare professionals [23]. For example, less than 5% of the datasets used in AI healthcare applications are sourced from low-resource settings, raising concerns about the generalizability and fairness of AI models [24]. The lack of local expertise is another major challenge: the State of AI Report 2021 reveals that a significant proportion of AI professionals are concentrated in North America, Europe, and East Asia, leaving other regions underrepresented [25].

The disparities in global AI investments and research funding are significant, particularly concerning regions like Africa and Latin America. In the fourth quarter of 2023, the United States accounted for over 50% of global funding for AI startups, with Asia and Europe together commanding nearly the same amount of funding strength. In contrast, regions such as Africa and Latin America received a minimal share of these investments [26]. Furthermore, the allocation of research funding to low- and middle-income countries (LMICs) is disproportionately low. For instance, an analysis revealed that as little as 0.2% (USD 48 million) of approximately USD 21.4 billion in grant funding for noncommunicable diseases was allocated to institutions in LMICs, despite these countries bearing a significant burden of such diseases [27]. These statistics underscore the challenges faced by Africa and Latin America in securing adequate funding for AI development and healthcare research, highlighting the need for a more equitable distribution of resources to address global health disparities effectively.

These challenges are further exacerbated by socioeconomic inequalities, regulatory hurdles, and concerns over data privacy and security [28]. For instance, inconsistent or absent data protection laws in many LMICs limit the ability to collect and share data safely, hindering collaborative efforts for AI model development [20]. Addressing these issues requires global partnerships and initiatives that focus on capacity building, equitable funding allocation, and the development of inclusive AI solutions tailored to local contexts.

Addressing these challenges requires a collaborative and inclusive approach that bridges the technological divide. International partnerships are essential in fostering knowledge exchange, enabling low-resource settings to benefit from the expertise and advancements of more technologically advanced nations. Such collaborations can support capacity building through training programs, workshops, and mentorship initiatives aimed at equipping local professionals with the skills needed to implement and adapt AI-driven healthcare solutions. Furthermore, partnerships can facilitate technology transfer by adapting AI tools to local contexts, ensuring they address region-specific health challenges. By sharing best practices and co-creating solutions, international alliances can accelerate the equitable deployment of AI in healthcare, ultimately contributing to global efforts toward achieving the Sustainable Development Goals (SDGs), particularly SDG 3 on good health and well-being.

## 3. International Partnerships for AI-Driven Healthcare

Note on Methodology: The international initiatives highlighted in this paper were identified through a targeted review of peer-reviewed publications, policy reports, and institutional websites published between 2020 and early 2024. Selection criteria included geographic diversity, relevance to AI in healthcare, documented multi-stakeholder collaboration, and evidence of impact or scalability. Where possible, initiatives supported by intergovernmental organizations (e.g., WHO, UN agencies), academic–industry consortia, or large-scale public programs were prioritized. While this is not a systematic review, efforts were made to reduce selection bias and provide a representative overview of global efforts in AI-driven healthcare collaboration.

### 3.1. Technology Transfer and Capacity Building

Partnerships between developed and developing countries play a crucial role in facilitating the transfer of AI technologies and expertise, thereby narrowing the global gap in AI adoption for healthcare [20]. For instance, collaborations between academic institutions in high-income and low-income countries help build local capacity in AI by providing access to resources, training, and technical mentorship. One notable example is the partnership between Imperial College London and universities in sub-Saharan Africa [29], focusing on training healthcare professionals in AI and data science to address pressing local healthcare challenges, such as infectious disease outbreaks and maternal health. By co-developing AI-driven solutions, this collaboration ensures that the technologies are contextually relevant and can effectively address regional needs. Similarly, the MIT Abdul Latif Jameel Clinic for Machine Learning in Health has partnered with institutions in South Asia to provide training programs and resources for the implementation of AI solutions in areas such as early disease detection and healthcare logistics [30]. These partnerships emphasize not only the transfer of technology but also the creation of sustainable ecosystems by enabling local researchers and practitioners to take ownership of AI projects. For example, these initiatives have empowered local healthcare providers to develop predictive models for disease outbreaks using region-specific data, significantly improving response times during crises.

Furthermore, international organizations such as the World Bank and the United Nations have launched capacity-building initiatives, providing funding and technical assistance to LMICs for implementing AI solutions tailored to their healthcare systems [31,32]. However, successful technology transfer requires more than just funding and training; it also necessitates addressing systemic challenges such as the lack of digital infrastructure, language barriers, and the adaptation of AI tools to local regulatory frameworks and cultural contexts [20,23]. By fostering equitable collaborations, these partnerships ensure that AI-driven healthcare solutions are not only transferred but also effectively integrated into local systems, bridging global healthcare disparities. Another example is the collaboration between Google Health and the Ministry of Health in India [33], where Google Health has provided AI tools for detecting diabetic retinopathy. This partnership includes training local healthcare workers to use the AI tools, ensuring that the technology is effectively integrated into the healthcare system. Such initiatives demonstrate the importance of capacity building alongside technology transfer to create sustainable healthcare solutions. In addition, the World Health Organization (WHO) has launched initiatives like the AI for Health focus group, which brings together stakeholders from different countries to share knowledge and best practices. This initiative aims to build capacity in developing countries by providing access to AI tools and training programs that enhance the ability of local healthcare systems to adopt these technologies [34].

### 3.2. Data Sharing and Ethical Considerations

AI systems require large, diverse datasets to achieve high performance and generalizability [35]. However, collecting such datasets often poses significant challenges, particularly in low-resource settings where data collection infrastructure is limited. International collaborations offer a promising avenue for addressing these challenges by enabling the pooling of data from different regions, thereby creating comprehensive and high-quality datasets. For example, the Global Alliance for Genomics and Health (GA4GH) is an international initiative that unites stakeholders from healthcare, research, and industry to develop frameworks for the ethical and secure sharing of genomic and clinical data across borders [36]. These frameworks address critical issues such as patient privacy, data security, and equitable access, ensuring that data sharing complies with ethical guidelines and local regulations.

Such collaborative efforts not only support the development of robust AI models that are representative of diverse populations but also help reduce algorithmic biases that can arise from underrepresented groups in datasets. For instance, GA4GH has supported projects that include genomic data from underrepresented populations, leading to improved diagnostic accuracy and better-tailored treatments for rare diseases. Similarly, regional data-sharing initiatives, like the European Health Data Space (EHDS) [37], aim to enable cross-country access to health data, fostering innovation in AI-driven healthcare solutions. By leveraging diverse datasets, these initiatives improve the reliability and fairness of AI models, ultimately enhancing healthcare outcomes on a global scale. Furthermore, these collaborations highlight the importance of capacity building in data governance. Ensuring responsible data sharing requires not only technical frameworks but also training programs to equip healthcare professionals and researchers with the skills to handle and interpret data responsibly. Addressing these challenges through international partnerships fosters an environment where AI can be both innovative and ethically grounded, paving the way for equitable and effective healthcare advancements. Another example is the Personal Health Train (PHT) initiative [38], which aims to enable secure and ethical data sharing without transferring raw data. The PHT uses federated learning, allowing AI models to be trained across multiple institutions while keeping the data locally stored. This approach addresses privacy concerns and complies with data protection regulations, such as the General Data Protection Regulation (GDPR) in the European Union, ensuring that data sharing is both effective and ethical. In Africa, the African Health Data Initiative (AHDI) has been playing a pivotal role in improving data-sharing frameworks and strengthening the capacity of local institutions to contribute to and benefit from global health datasets. Recognizing the barriers that African nations face in participating in international AI research—such as limited infrastructure, a shortage of skilled professionals, and concerns about data sovereignty—AHDI provides targeted support to address these challenges. By offering training programs on data governance, ethical considerations, and compliance with international standards, such as the General Data Protection Regulation (GDPR) and the Health Insurance Portability and Accountability Act (HIPAA), AHDI ensures that African nations can engage meaningfully in global AI collaborations while safeguarding patient privacy [39]. Furthermore, AHDI promotes the development of interoperable health data systems across African nations, enabling a more seamless integration of regional datasets into global initiatives. For example, the initiative has facilitated the adoption of standardized data-sharing protocols, ensuring that healthcare data collected in Africa can contribute to the development of AI models that are representative of diverse populations [40]. Through its efforts, AHDI also addresses the ethical implications of data sharing, advocating for equitable benefits from AI research and ensuring that African countries retain ownership of their health data while leveraging its value for local healthcare improvements. By fostering these frameworks and empowering local institutions, AHDI not only enables African nations to participate in international AI research but also helps to mitigate global healthcare disparities, ensuring that AI-driven solutions are inclusive and equitable.

Despite these initiatives, significant challenges persist, particularly regarding differing national regulations on data privacy, security, and governance, which can hinder effective and seamless data sharing across borders. For example, the General Data Protection Regulation (GDPR) in the European Union imposes strict rules on the storage, transfer, and use of personal data, ensuring high levels of privacy and security. However, these regulations often conflict with or exceed the standards of other countries, creating barriers to cross-border collaborations [41]. Additionally, some low- and middle-income countries (LMICs) lack comprehensive data protection frameworks, further complicating efforts to share healthcare data internationally [42]. Such discrepancies can slow down the development of global AI models and exacerbate existing inequities in AI-driven healthcare [43].

To address these challenges, the World Health Organization (WHO) has been spearheading efforts to develop global standards and guidelines for the ethical use of AI and data sharing in healthcare [20]. For instance, the WHO Guidance on Ethics and Governance of Artificial Intelligence for Health outlines principles such as transparency, accountability, and fairness, providing a framework for the responsible use of AI in healthcare settings. These standards aim to create a common framework that respects regional regulations while enabling international collaboration, ensuring that ethical considerations are not compromised in the pursuit of technological advancement. Furthermore, the WHO emphasizes the importance of capacity building in countries with underdeveloped regulatory systems, helping them establish robust legal and institutional frameworks that align with global standards.

In addition to WHO’s efforts, multi-stakeholder organizations like the International Telecommunication Union (ITU) and the Global Alliance for Genomics and Health (GA4GH) are also working on harmonizing data-sharing protocols [44]. These initiatives focus on creating interoperable systems that can operate within the constraints of varying regulations, ensuring secure and equitable access to healthcare data for AI research [45]. By fostering collaboration among governments, researchers, and private entities, these frameworks aim to bridge regulatory gaps, mitigate legal conflicts, and unlock the full potential of international data sharing for AI-driven healthcare.

### 3.3. Case Study: Global Health Initiatives

One notable example of an international AI partnership is the RAD-AID initiative, which focuses on improving access to radiology services in underserved regions by leveraging AI technologies [46]. Recognizing the critical shortage of radiologists in many low- and middle-income countries (LMICs), RAD-AID collaborates with local healthcare providers to enhance diagnostic capabilities and address healthcare disparities. In Tanzania, RAD-AID has partnered with local hospitals to deploy AI-driven radiology solutions, specifically targeting conditions such as tuberculosis and pneumonia, which are prevalent in the region. Through the use of AI tools that analyze chest X-rays and other medical images, the initiative has significantly enhanced the efficiency and accuracy of diagnostic workflows.

Moreover, RAD-AID emphasizes capacity building by providing hands-on training to local healthcare workers and radiologists on how to effectively use and interpret AI outputs. For example, healthcare workers are trained not only in operating AI-powered systems but also in understanding their limitations and integrating them into clinical decision-making processes. This dual focus on technology and education ensures that the solutions are sustainable and contextually relevant, empowering local staff to take ownership of the systems. The impact of this partnership extends beyond radiology; by improving diagnostic infrastructure, the initiative has also strengthened referral pathways and enabled better treatment planning for diseases like cancer, tuberculosis, and cardiovascular conditions. RAD-AID’s work in Tanzania exemplifies the potential of AI to transform healthcare in resource-limited settings by bridging critical gaps in medical expertise and infrastructure.

Another example is the AI4COVID initiative [47], a global collaboration involving multiple countries, including the United States, Italy, and India, aimed at leveraging artificial intelligence (AI) to combat the COVID-19 pandemic through advanced medical imaging. This initiative brought together researchers, healthcare professionals, and policymakers from diverse backgrounds, fostering a multidisciplinary approach to address the pressing challenges posed by the pandemic. By pooling resources, expertise, and datasets across borders, AI4COVID enabled the rapid development of AI models capable of detecting COVID-19-related abnormalities in chest X-rays and CT scans with high accuracy. The initiative was particularly impactful in low-resource settings, where shortages of trained radiologists and diagnostic equipment often hinder the timely detection and management of diseases. AI4COVID’s tools bridged these gaps, offering scalable, automated solutions that enhanced diagnostic efficiency and accuracy. For example, these AI-driven systems were integrated into clinical workflows to assist frontline healthcare workers, providing reliable diagnostic support in regions with limited healthcare infrastructure. In addition to diagnostics, AI4COVID also focused on resource allocation and disease monitoring. AI models developed through the initiative were used to predict disease progression, enabling healthcare systems to better prioritize patients and allocate critical resources such as ICU beds, oxygen supplies, and ventilators. Moreover, the models supported large-scale screening efforts, helping governments and health organizations track the spread of the virus more effectively.

Crucially, AI4COVID underscored the importance of data diversity and ethical considerations in AI research. By collecting datasets from diverse populations across participating countries, the initiative enhanced the robustness and generalizability of its models while addressing biases that often arise from limited or homogenous data. Collaborative efforts also tackled cross-border challenges related to data privacy and security, ensuring compliance with local regulations while fostering trust among stakeholders. Such initiatives demonstrate the transformative potential of international cooperation in harnessing AI for global health challenges. By accelerating the development and deployment of AI solutions, AI4COVID not only contributed to the immediate fight against COVID-19 but also laid the groundwork for more resilient healthcare systems capable of addressing future pandemics and health emergencies.

The AI-powered Maternal and Child Health (MCH) initiative [48] is another compelling example that underscores the importance of global health partnerships in leveraging technology to address critical healthcare challenges. Led by UNICEF in collaboration with AI researchers and institutions from multiple countries, this initiative aims to improve maternal and child health outcomes, particularly in low-income and underserved regions. By harnessing AI to analyze large volumes of health data—such as antenatal records, demographic information, and environmental factors—the initiative predicts potential complications during pregnancy and childbirth, enabling healthcare workers to take timely preventive actions. In rural areas where access to skilled obstetricians and medical infrastructure is limited, AI-powered tools have proven to be transformative. These tools are integrated into mobile applications and portable diagnostic devices, providing community health workers with actionable insights to identify high-risk pregnancies and refer patients to nearby facilities for advanced care. For example, AI algorithms have been used to predict conditions such as preeclampsia, gestational diabetes, and preterm labor, significantly improving the ability to address these conditions before they become life-threatening.

Beyond diagnostics, the initiative also focuses on improving healthcare delivery through better resource allocation. AI models assist governments and NGOs in optimizing the distribution of essential supplies like prenatal vitamins, vaccines, and emergency medical equipment, ensuring that resources reach the communities most in need. Additionally, the MCH initiative emphasizes capacity building by training local healthcare workers to use AI tools effectively while ensuring that these technologies align with ethical standards and local cultural contexts.

The results have been impactful: pilot programs have shown reductions in maternal and neonatal mortality rates in regions where the initiative has been implemented. Moreover, the initiative has sparked broader discussions about the role of AI in addressing health inequities and building resilient healthcare systems in resource-limited settings. This collaborative effort demonstrates the potential of AI not only to improve health outcomes but also to empower local healthcare systems through technology transfer, capacity building, and knowledge sharing.

These case studies illustrate the significant impact of international AI partnerships on improving healthcare in underserved regions. By pooling resources, sharing expertise, and leveraging cutting-edge technology, such collaborations demonstrate how AI can be harnessed to address critical healthcare challenges and promote equitable access to care.

## 4. Challenges and Opportunities

While international partnerships offer significant opportunities to advance AI-driven healthcare, they also face a range of challenges that must be addressed to achieve their full potential and contribute to the UN Sustainable Development Goals (SDGs). Key among these challenges are differences in regulatory frameworks, data privacy concerns, and the persistent digital divide. Regulatory inconsistencies—such as varying standards for data protection, ethical considerations, and AI governance—can create barriers to cross-border collaboration and hinder the sharing of critical healthcare data. For instance, stringent data privacy laws like the European Union’s General Data Protection Regulation (GDPR) may conflict with less comprehensive regulations in other regions, complicating the development of global datasets and AI models [49]. These regulatory disparities present a challenge to SDG 3 (Good Health and Well-being), as they prevent the efficient sharing of health data across borders, limiting the potential for AI to address global health challenges.

Harmonizing regulations across countries is essential to enable seamless data sharing and collaborative research, directly contributing to SDG 9 (Industry, Innovation, and Infrastructure). Developing standardized protocols for AI implementation, such as frameworks for data anonymization, interoperability, and algorithm transparency, can help establish a common foundation for international partnerships. These protocols would not only ensure compliance with local regulations but also build trust among stakeholders, fostering a secure and equitable environment for AI adoption [50]. By creating a regulatory framework conducive to innovation, countries can accelerate the development and deployment of AI healthcare technologies, ensuring that the benefits are accessible to all.

Additionally, addressing the digital divide is critical for ensuring that the benefits of AI-driven healthcare are distributed equitably, aligning with SDG 10 (Reduced Inequality). Many low- and middle-income countries (LMICs) lack the infrastructure needed to support advanced AI technologies, such as high-speed internet, cloud computing, and modern healthcare facilities. Investments in healthcare infrastructure—such as telemedicine platforms, diagnostic tools, and electronic health records—are necessary to bridge these gaps. Equally important are digital literacy programs aimed at equipping healthcare workers with the skills to effectively use AI tools. These programs should focus on both technical training and ethical considerations, ensuring that healthcare professionals are not only proficient in using AI but also understand its limitations and implications [51]. By empowering healthcare workers in LMICs with the necessary skills and infrastructure, international partnerships can help reduce inequalities and ensure that AI healthcare benefits all populations.

Moreover, fostering partnerships that prioritize capacity building in under-served regions can further mitigate disparities and advance SDG 3. For example, international collaborations can include provisions for technology transfer, funding for local research, and the development of region-specific AI models that cater to local healthcare challenges. By addressing these barriers, international partnerships can unlock the full potential of AI in healthcare, promoting equitable access to innovative solutions and advancing global health outcomes, thus contributing to the realization of SDGs across the globe.

While international collaborations offer immense promise, several persistent challenges must be addressed to ensure that AI in healthcare is inclusive, effective, and sustainable. These challenges go beyond regulatory misalignment and digital divides. They also include structural barriers such as the lack of localized datasets and AI talent in LMICs, ethical concerns around algorithmic bias and consent, uncertainties in long-term project sustainability, and the often limited engagement of local communities in AI model development. Moreover, the rapid pace of innovation often outpaces policy, creating legal and operational uncertainties. Addressing these issues requires not only multilateral cooperation but also locally grounded strategies that reflect regional contexts, needs, and values.

## 5. Conclusions

Artificial Intelligence (AI) has emerged as a transformative force in global healthcare, with the potential to bridge gaps in access, quality, and efficiency—especially when deployed through inclusive international partnerships. This article highlights how collaborations that emphasize technology transfer, ethical data sharing, and capacity building can help ensure that AI innovations benefit both high-resource and low-resource settings. Such partnerships are instrumental in advancing the United Nations Sustainable Development Goals (SDGs), particularly SDG 3 (Good Health and Well-being), SDG 9 (Industry, Innovation, and Infrastructure), and SDG 10 (Reduced Inequality). However, realizing the full impact of AI in healthcare requires addressing persistent challenges, including regulatory fragmentation, the digital divide, and limited representation of low- and middle-income countries in AI development. A coordinated, cross-border approach that integrates technical advancement with ethical governance and equitable resource distribution is therefore essential. By aligning innovation with global solidarity, international cooperation in AI-driven healthcare can help build more resilient and inclusive health systems worldwide.

While the findings in this article are based on a narrative synthesis of high-profile initiatives rather than experimental evaluation, they offer actionable insights into how international partnerships can shape the future of AI in global healthcare. The highlighted initiatives—though not exhaustive—collectively reveal patterns of effective collaboration: capacity building through training, ethical data sharing through federated models, and equitable technology transfer mechanisms. These findings suggest that a deliberate, partnership-driven strategy is essential for translating AI advancements into scalable, inclusive healthcare solutions. Future research may build on this foundation by conducting systematic evaluations of such initiatives to assess their long-term impact and replicability.

## Data Availability

Not applicable.
